# Commentary: Beyond distal anastomosis new entry: Distal re-entry tears as well

**DOI:** 10.1016/j.xjtc.2021.06.023

**Published:** 2021-06-23

**Authors:** Thomas M. Beaver, Prashanth Vallabhajosyula

**Affiliations:** a Division of Cardiovascular Surgery, University of Florida College of Medicine, Gainesville, Fla; b Yale Aortic Institute, Yale University, New Haven, Conn

**Central Message**

Although distal anastomosis new entry tear occurs following acute type A aortic dissection repair, further distal re-entry tears also contribute to persistent false lumens.

**Figure:**
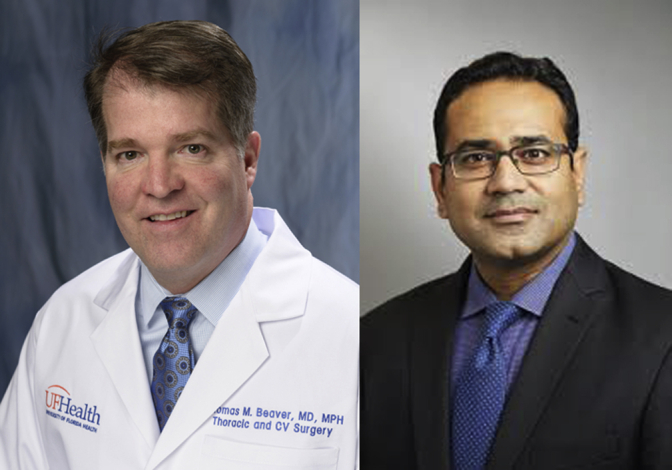
Thomas M. Beaver, MD, MPH, and Prashanth Vallabhajosyula, MD, MS

See Article page 1.

A false lumen can persist following acute type A aortic dissection (ATAAD) repair either from the complex aortic dissection itself or from incomplete suture apposition resulting in distal anastomosis new entry tear, as highlighted in the current report.^1^ Although subsequent aneurysmal dilation of the arch and proximal descending aorta following hemiarch repair of ATAAD does occur, the safety of secondary repair has been established at aortic centers.^2^

In an effort to prevent any reoperations, many aortic surgeons have adopted an increasingly more aggressive approach to ATAAD, employing total arch replacement often combined with a frozen elephant trunk repair.^3^ This can be a complex undertaking in the setting of ATAAD and has a known paraplegia rate of 4% globally.^4^ Furthermore, higher stroke rate with total arch replacement compared with hemiarch repair is reported, suggesting that this approach has to be carefully adopted.^5^ Accordingly, a more limited arch replacement to zone 2 with arch debranching as described by Desai and colleagues^6^ has been employed at many centers. The zone 2 approach sets patients up for subsequent reintervention with a less-invasive thoracic endovascular aortic repair (TEVAR) strategy using either a branched device into the left subclavian artery or a carotid-subclavian bypass and standard TEVAR into a debranched zone 0.

Again, all of the above approaches can be a daunting for cardiac surgeons who repair 1 to 3 dissections a year. Hence the novelty of the current report, where the authors share their concept of employing a spiral stent Ascyrus Medical Dissection Stent (AMDS) (CryoLife Inc, Kennesaw, Ga) at the time of hemiarch repair of ATAAD in an effort to not only prevent distal anastomosis new entry tear, but also maintain true lumen integrity and mitigate the problem of a persistent false lumen in the arch and beyond with its inherent risks of malperfusion and/or aneurysmal degeneration.^1^ The experience with the AMDS device is limited and although there was 100% successful device deployment and positive remodeling in a midterm report of 46 ATAAD patients, there was still a persistent false lumen in the arch of 10 out of 39 (26%) patients and in the proximal descending aorta in 18 out of 38 (47%).^7^

A similar strategy has been previously reported in acute type B aortic dissection: The provisional extension to induce a complete attachment technique, which includes covering the primary tear with a covered TEVAR stent and then employing bare metal stents in the remainder of the visceral abdominal aorta to the iliac bifurcation (Zenith Endovascular Dissection System, Cook Medical, Bloomington, Ind). However, in a trial of complicated type B dissections, a partially thrombosed false lumen was noted in 31 out of 39 (79.5%) patients at 12 months and 15 out of 39 (38.5%) had an increase of >5 mm in their aortic diameter.^8^ Unfortunately, dissections are a complex entity and distal re-entry tears beyond suture lines may hamper the provisional extension to induce a complete attachment and AMDS strategies.

Another concern is future reoperative surgery. In contrast to patients with a total arch repair or even a zone 2 repair, in patients with AMDS surgeons would have to deal with any residual stent material in the arch. Accordingly, hemiarch repair alone for ATAAD remains a valid strategy for the vast majority of surgeons who rarely encounter them.
